# Evaluation of Colistin Susceptibility of *Klebsiella pneumoniae* Strains Exposed to Rotating Magnetic Field

**DOI:** 10.3390/ijms26178281

**Published:** 2025-08-26

**Authors:** Agata Pruss, Dagmara Kobylińska, Karol Fijałkowski, Helena Masiuk, Paweł Kwiatkowski

**Affiliations:** 1 Department of Laboratory Medicine, Pomeranian Medical University in Szczecin, Powstancow Wlkp. 72, 70-111 Szczecin, Poland; dagmara.kobylinska97@gmail.com; 2 Department of Microbiology and Biotechnology, West Pomeranian University of Technology in Szczecin, Piastow 45, 70-311 Szczecin, Poland; karol.fijalkowski@zut.edu.pl; 3 Department of Clinical Microbiology, Pomeranian Medical University in Szczecin, Powstancow Wlkp. 72, 70-111 Szczecin, Poland; helena.masiuk@pum.edu.pl; 4 Department of Diagnostic Immunology, Pomeranian Medical University in Szczecin, Powstancow Wlkp. 72, 70-111 Szczecin, Poland; pawel.kwiatkowski@pum.edu.pl

**Keywords:** antibiotic resistance, colistin, rotating magnetic field, *Klebsiella pneumoniae*

## Abstract

*Klebsiella pneumoniae*, due to its capacity to produce numerous virulence factors and form biofilms, is one of the most significant etiological agents of nosocomial infections. The extensive and often unwarranted use of antibiotic therapy has driven the emergence of various mutations, adaptive mechanisms, and horizontal gene transfer among *K. pneumoniae* strains, resulting in resistance to most beta-lactam antibiotics, carbapenems, and the last-resort drug—colistin. A promising alternative or adjunctive treatment is the application of rotating magnetic fields (RMFs). The present study aimed to evaluate changes in colistin susceptibility among 20 extended-spectrum beta-lactamases (ESBLs) and 20 *K. pneumoniae* carbapenemase (KPC)-positive *K. pneumoniae* strains isolated from hospital infections following exposure to RMF at frequencies of 5 and 50 Hz. Exposure to RMF at 5 Hz resulted in decreased colistin minimum inhibitory concentration (MIC) values in over half of the tested (ESBLs) and (KPC)-positive strains. Additionally, RMF at 50 Hz reduced colistin MIC values in 30% of (ESBL)-positive and 40% of (KPC)-positive strains. Therefore, in the future, RMF may be developed as a supportive therapeutic strategy to improve the efficacy of antibiotics in the treatment of infections caused by multidrug-resistant (MDR) pathogens, including colistin-resistant *K. pneumoniae.*

## 1. Introduction

*Klebsiella pneumoniae* is classified as an opportunistic microorganism due to its pathogenic potential, which becomes particularly evident in various forms of immunosuppression or when the bacterium translocates from its natural habitat to other body sites [1]. It is among the most frequently isolated pathogens from hospital-acquired infections [2]. Two major pathotypes of *K. pneumoniae* have been identified based on their virulence: classical (*cKp*) and hypervirulent (*hvKp*) strains [3]. The rising antibiotic resistance observed in *hvKp* strains has rendered them increasingly life-threatening.

Despite extensive research into the pathogenic mechanisms, virulence factors, and clinical progression of *K. pneumoniae* infections, significant gaps in understanding remain. Genetic studies of *K. pneumoniae* are expected to gain importance, with the aim of elucidating the underlying pathophysiology and facilitating the development of effective non-antibiotic therapies to reduce infection rates [4].

The organism’s extensive multidrug resistance has led to its designation as a high-priority alert pathogen. According to the European Centre for Disease Prevention and Control (ECDC), several *K. pneumoniae* strains have been classified as pandrug-resistant (PDR), extensively drug-resistant (XDR), or multidrug-resistant (MDR) due to their resistance to nearly all currently available antibiotics [5].

The emergence of carbapenem resistance in *K. pneumoniae* has become a major public health concern in recent years. This resistance is primarily driven by horizontal gene transfer, particularly via plasmid exchange between different bacterial strains or species [6]. *K. pneumoniae* is a prominent producer of carbapenemases, including *K. pneumoniae* carbapenemase (KPC) and New Delhi metallo-β-lactamase (NDM). Additionally, carbapenem-hydrolyzing class D β-lactamases (CHDLs), which are chromosomally encoded in *Acinetobacter* species, have been acquired by *K. pneumoniae* through interspecies gene transfer.

Initially, members of the *Enterobacteriaceae* family exhibited limited resistance to carbapenems. However, the incorporation of CHDLs, in combination with other resistance determinants, such as efflux pump overexpression and porin mutations, has contributed to the emergence of specific variants, notably OXA-48-type carbapenemases, now commonly associated with *K. pneumoniae* [7,8]. Carbapenemase production in *K. pneumoniae* frequently co-occurs with resistance to multiple other antibiotic classes, further complicating treatment strategies.

In Poland, approximately 90% of *K. pneumoniae* strains produce extended-spectrum β-lactamases (ESBLs), enzymes capable of hydrolyzing not only cephalosporins but also cephamycins, penicillins, and monobactams [9,10]. Although ESBL activity is theoretically inhibited by β-lactamase inhibitors such as sulbactam, clavulanic acid, or tazobactam, many ESBL-producing strains also exhibit resistance to these agents. While the detection of an ESBL-producing strain may suggest the potential efficacy of carbapenems, this does not preclude resistance to other antimicrobial classes.

Colistin, a polymyxin-class antibiotic, is known for its significant toxicity, particularly neurotoxic and nephrotoxic effects, which led to its previous withdrawal from routine clinical use. However, the resurgence of infections caused by multidrug-resistant (MDR) *Enterobacteriaceae* (particularly strains susceptible only to colistin) has led to its reintroduction as a therapeutic option [11]. Today, colistin is considered a last-resort antibiotic for the treatment of *K. pneumoniae* infections that produce carbapenemases [12].

Structurally, colistin consists of a cyclic peptide linked to a fatty acid chain, imparting both hydrophilic and hydrophobic properties. This amphipathic nature facilitates binding to the phospholipids in the bacterial outer membrane, disrupting magnesium and calcium ion homeostasis. Such disruption compromises membrane integrity, increases permeability, and enhances antibiotic uptake into the bacterial cell [12].

Originally, *K. pneumoniae* lacked intrinsic resistance to colistin. Resistance has since emerged, primarily via chromosomal mutations, notably involving overexpression or inactivation of the *mgrB* gene. These genetic changes alter the structure of lipopolysaccharides (LPSs) and activate efflux pumps, thereby reducing intracellular antibiotic concentrations. Additionally, it has been hypothesized that colistin resistance in some *K. pneumoniae* strains may have arisen through horizontal gene transfer of plasmids carrying the *mcr* gene from other bacterial species [13].

The World Health Organization (WHO) has designated *K. pneumoniae* as a priority pathogen requiring urgent attention due to its increasing resistance to current treatment options. This recognition has catalyzed global research efforts aimed at developing innovative and effective therapeutic strategies to combat infections caused by this organism [14]. Phytotherapy, for example, is also under investigation, particularly the use of essential oils with well-documented bactericidal and bacteriostatic properties, offering a natural approach to infection control [15].

In recent years, there has been an increasing interest in understanding how magnetic fields affect the proliferation and life cycle of both eukaryotic and prokaryotic cells. Researchers are particularly focused on the impact of these fields on microorganisms, especially pathogenic varieties. Experimental observations have been made regarding their responses to rotating magnetic fields (RMFs), driven partly by the need to explore alternative therapeutic methods for treating infections caused by MDR strains. Notably, the application of variable frequency RMF, with carefully controlled parameters, has been shown to influence various biological processes, including metabolic and enzymatic activity, biofilm formation, growth rates, cell viability, ion transport, genetic material synthesis and transcription, and even the lysis of pathogenic species and strains [16]. In our previous research, we demonstrated that a specific type of magnetic field, known as the RMF, influences charged molecules, such as antimicrobial ions, by aligning their movement with the rotation of the magnetic field. Additionally, we showed that RMF has varying effects—ranging from negative to positive—on the growth, metabolic activity, and biofilm formation of various microbial species and strains, such as methicillin-resistant *Staphylococcus aureus*—MRSA [17]. In a separate investigation, we examined the combined effects of RMF and antimicrobials (antibiotics and antiseptics) on *S. aureus* and *P. aeruginosa* biofilms. The results revealed that biofilms treated with both RMF and antimicrobials exhibited a 50% greater reduction compared to those treated with antimicrobials alone. Furthermore, we established that RMF enhances the bactericidal efficacy of different antibiotic classes against *S. aureus*, particularly methicillin-resistant strains (MRSA). This observed enhancement was most pronounced with antibiotics that target and disrupt bacterial cell wall structures [17,18,19]. The present study aimed to evaluate changes in drug susceptibility to colistin of 20 extended-spectrum beta-lactamases (ESBLs) and 20 *K. pneumoniae* carbapenemase (KPC)-positive *K. pneumoniae* strains isolated from hospital infections using RMF of 5 and 50 Hz.

## 2. Results

### 2.1. PFGE Results

**Figure 1:**
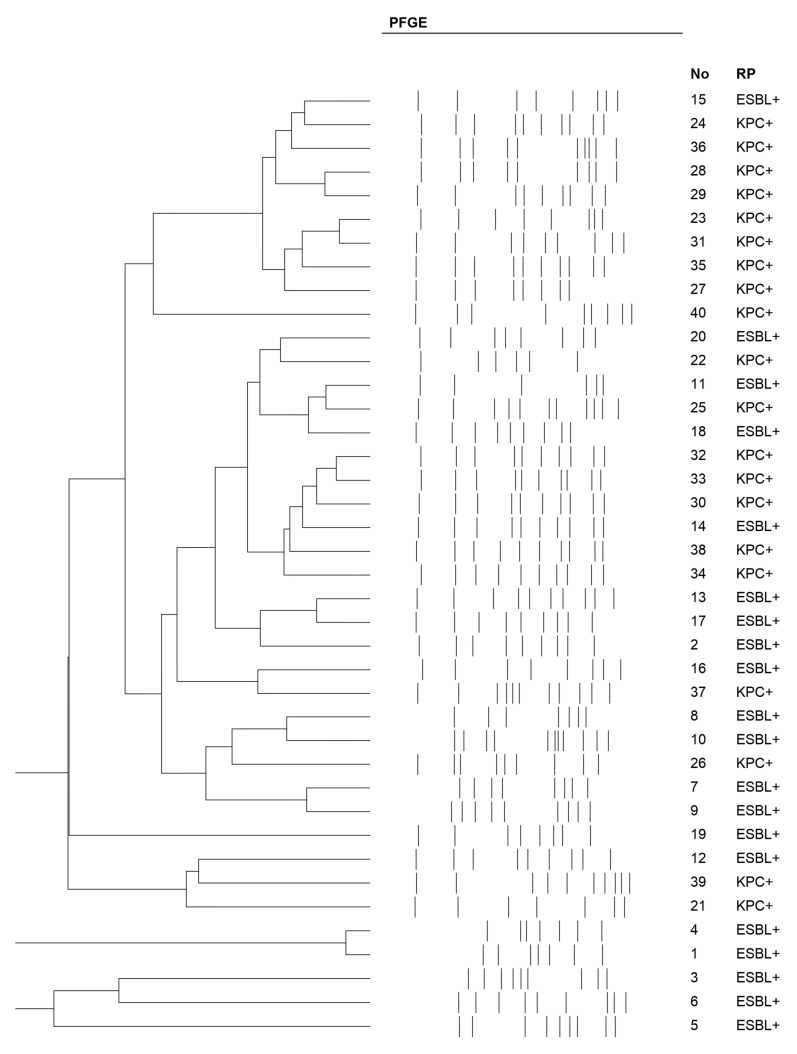
Pulsed-field gel electrophoresis (PFGE) of the strains.

All *K. pneumoniae* strains included in this study were clinical isolates, each representing a unique PFGE pulsotype. This deliberate selection of genetically diverse strains ensured broad representation across different lineages (Figure 1).

### 2.2. Drug Susceptibility of (ESBLs)-Positive K. pneumoniae

The tested strains exhibited significant resistance to the majority of antibiotics within the beta-lactam group, as well as to combinations of beta-lactams with beta-lactamase inhibitors, due to the ESBL mechanism. In contrast, a low level of resistance was noted for amikacin, gentamicin, ciprofloxacin, trimethoprim, and sulfamethoxazole. Notably, full susceptibility to ertapenem, meropenem, and imipenem was observed (Table 1, Supplementary Materials—Table S1).

### 2.3. Drug Susceptibility of (KPC)-Positive K. pneumoniae

The tested strains exhibited significant resistance to most antibiotics evaluated, attributed to the KPC mechanism. Detailed data are listed in Table 2 and Supplementary Materials—Table S1.

### 2.4. Quantitative Method for the MIC Determination of Colistin

All tested strains exhibited resistance to colistin, both among (ESBL)-positive and (KPC)-positive groups. The E-test for colistin yielded identical MIC values for all strains within the KPC group. In contrast, six strains in the ESBL group showed a one-value discrepancy in MIC compared to the quantitative method.

### 2.5. Susceptibility to Colistin After RMF Exposure

At a frequency of 5 Hz, 55.0% of *K. pneumoniae* (ESBL)-positive strains exhibited no change in the minimum inhibitory concentration (MIC) compared to the control. Conversely, a reduction in colistin MIC was noted in 45% of the strains. The results of the two methods for determining colistin MIC (E-test and dilution method) were largely consistent. However, due to the differing ranges of MIC values between the two assays, the E-test indicated a change in post-exposure MIC from 6 to 4 for four strains, while the dilution method showed a decrease from 8 to 4. In both instances, the observed change was a reduction in post-exposure MIC. The most significant reduction in MIC values across both methods (from 16 to 4) was recorded for a single isolate (Table 3). Half of the *K. pneumoniae* (KPC)-positive strains exhibited no change after exposure to 5 Hz RMF. The other half demonstrated a decrease in MIC by at least one value. Identical results were obtained for both methods used, as detailed in Table 3 and Supplementary Materials—Table S1.

When RMF was applied at 50 Hz, results varied. Among *K. pneumoniae* (ESBL)-positive strains in the dilution method, 60% and 50% in the E-test method showed no change in MIC values between control and test samples. 35% of strains in the dilution method and 25% in the E-test method showed a decrease in MIC values after exposure to RMF at this frequency. In addition, one isolate in the dilution method showed an increase in MIC values, while in the E-test method, an increase in MIC values was observed for three strains (Table 4).

In the group of *K. pneumoniae* (KPC)-positive strains, identical results were obtained using both methods (dilution and E-test). 55.0% showed no change in MIC values when comparing control and 50 Hz RMF-treated samples. 40% of the strains showed a decrease in MIC values. It is worth noting that one isolate experienced an increase in MIC values after being treated with 50 Hz RMF (Table 4, Supplementary Materials—Table S1).

**Figure 2:**
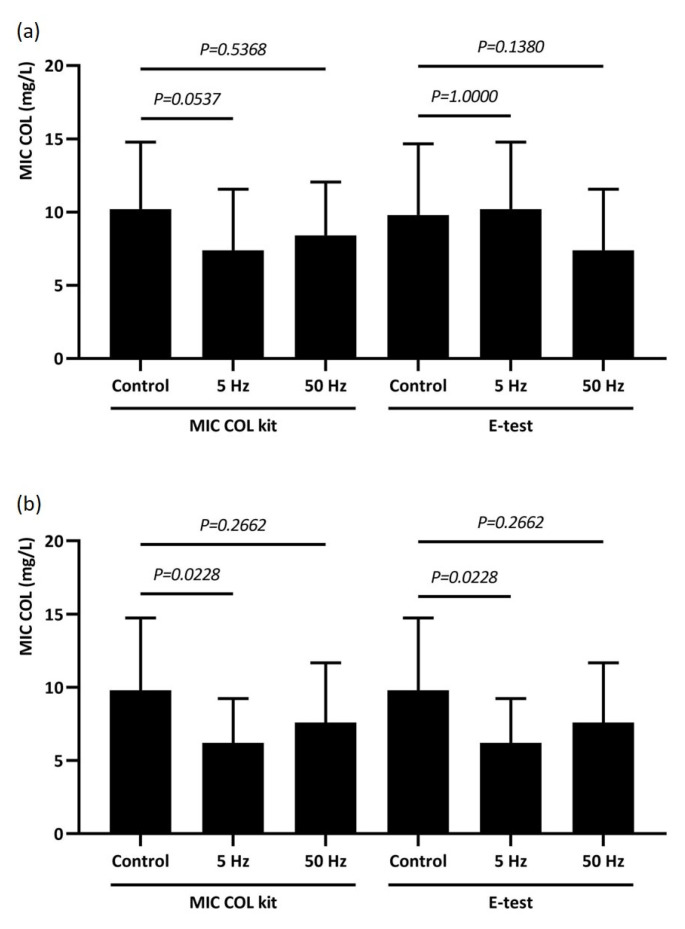
Comparison of the effects of two RMF frequencies on the MIC of colistin in the *K. pneumoniae* (ESBLs)-positive (**a**) and *K. pneumoniae* (KPC)-positive (**b**) groups.

Based on the tests performed, a decrease in the MIC of colistin (tested by the dilution method and E-test) was noted for ESBL strains compared to controls, although these results were not statistically significant (Figure 2a). Similar results were noted for KPC strains; however, only RMF 5 Hz resulted in a statistically significant decrease in COL MIC, tested by both E-test and dilution methods (*p* = 0.0228) (Figure 2b). A comparison of the effects of RMF on both groups of *K. pneumoniae* is shown in Figure 2.

## 3. Discussion

*K. pneumoniae* is a microorganism strongly associated with multidrug resistance, exhibiting rapid and complex adaptive mechanisms that render it nearly impervious to most antibiotic classes [14]. Increasing globalization and high levels of human mobility have facilitated the widespread dissemination of *K. pneumoniae* across diverse geographic regions, making effective containment extremely challenging. Clinically, this pathogen is frequently isolated in cases of severe pneumonia, urinary tract infections, bacteremia, sepsis, and wound infections, thereby posing a significant threat to patient safety. The complexity of its resistance mechanisms further exacerbates the clinical challenge, severely limiting available therapeutic options. In the most critical cases, particularly when infections are caused by multidrug-resistant strains, healthcare providers may be left with no viable antibiotic treatments, leading to potentially fatal outcomes [20,21].

As noted by Navon-Venezia et al., resistance of *K. pneumoniae* to penicillin was first documented in the literature as early as 1960 [21]. In 1985, Kliebe et al. reported the first isolation of an extended-spectrum β-lactamase (ESBL)-producing strain from a patient in Germany [22]. Livermore and Woodford later emphasized that the emergence of ESBL-type enzymes was an inevitable consequence of the frequent and often indiscriminate use of β-lactam antibiotics [23]. From the 1990s onward, (ESBL)-positive *K. pneumoniae* strains have accounted for nearly 60% of all hospital-acquired infections [24,25]. These strains produce enzymes capable of hydrolyzing a wide range of β-lactam antibiotics and exhibit variable susceptibility to other antimicrobial classes.

Data from the European Antibiotic Awareness Day report revealed a growing resistance trend among (ESBL)-producing *K. pneumoniae* strains to fluoroquinolones and aminoglycosides across European Union countries between 2009 and 2012 [26]. Supporting these findings, a study by Pakzad et al. reported that 77% of *K. pneumoniae* strains were resistant to ciprofloxacin, with 95% also resistant to cephalosporins, aminoglycosides, and selected β-lactams [27]. In our own study, we similarly observed complete resistance of (ESBLs)-positive strains to β-lactams, along with partial resistance to aminoglycosides, ciprofloxacin, and trimethoprim–sulfamethoxazole. However, many isolates retained susceptibility to carbapenems.

Carbapenems are among the most potent antibiotics available for the treatment of severe infections, particularly when their use is guided by antibiogram results. However, numerous clinical isolates of *K. pneumoniae* now produce enzymes with high hydrolytic activity, resulting in resistance to this class of antibiotics. The increasing prevalence of carbapenem-resistant *K. pneumoniae* presents a major clinical challenge, affecting both hospitalized patients and those in outpatient settings. This pathogen is a key producer of carbapenemases, which are classified into three main families: KPC, MBL (e.g., NDM), and OXA-48-like enzymes. These carbapenemases confer resistance not only to carbapenems but also to other antibiotic classes, including β-lactams, aminoglycosides, and fluoroquinolones [28].

The distribution of carbapenemase types varies significantly across geographic regions, with frequent shifts driven by the rapid dissemination of resistant strains. A study assessing the epidemiological situation in Poland revealed that the predominant carbapenemases produced by *K. pneumoniae* were NDM-type metallo-β-lactamase (71%), followed by KPC enzymes (29%) [29]. Recent studies indicate that in Poland, (NDM)-producing *K. pneumoniae* strains, particularly the ST11 NDM-1 lineage, are more prevalent than KPC producers, highlighting the urgent need for targeted surveillance and infection control measures [30,31]. In alignment with these findings, our research demonstrated complete resistance of the tested strains to all β-lactam antibiotics, along with high levels of resistance to aminoglycosides, ciprofloxacin, and trimethoprim–sulfamethoxazole.

Due to the extensive and advanced multidrug resistance exhibited by *K. pneumoniae* in the late 20th century, colistin was reintroduced as a last-resort antibiotic for treating infections caused by resistant strains [32,33]. Initially, *K. pneumoniae* lacked inherent resistance mechanisms to colistin, as the antibiotic had previously been withdrawn from clinical use due to its notable toxicity. Consequently, microorganisms had not developed widespread resistance to this drug. However, shortly after its reinstatement for treating infections resistant to other antibiotic classes, colistin-resistant *K. pneumoniae* strains began to emerge [34]. The increasing detection of such resistant strains across multiple regions presents a significant challenge for infection management worldwide [35,36]. In our study, all tested strains, including both (ESBL)-and (KPC)-positive isolates, demonstrated resistance to colistin, exhibiting markedly elevated minimum inhibitory concentrations (MICs).

The treatment of *K. pneumoniae* infections presents significant challenges due to the elevated risk of mortality and the complexity of its antibiotic resistance. This species possesses well-developed mechanisms conferring resistance to most, if not all, available antibiotics, alongside a rapid adaptability to environmental pressures. Moreover, *K. pneumoniae* is classified as an alert pathogen, distinguished by its high-level antibiotic resistance, rapid transmission, considerable epidemiological potential, and immediate threat to human health. Consequently, these factors have driven research efforts focused on identifying alternative and off-label therapeutic strategies for infections caused by the most virulent and drug-resistant *K. pneumoniae* strains. The literature presents various alternative methods that exhibit either a bacteriostatic or bactericidal effect against *K. pneumoniae*. These methods primarily aim to reduce the proliferation of multidrug-resistant strains and impede the development of resistance mechanisms. One notable approach involves the application of the RMF. According to Woroszyło et al., this method shows great promise, predicting high efficacy and versatility, as it could serve as an adjunct to antibiotic therapy or be utilized independently [37]. Research conducted by Nawrotek et al. and Hu et al. indicates that RMF can slow cell proliferation in the studied bacterial species [38,39]. Interestingly, RMF may also stimulate growth, particularly among Gram-positive bacteria, as evidenced by findings from Fijałkowski et al. [17]. In a separate investigation, the same research group examined the effects of exposure duration to RMF, along with variations in magnetic induction and frequency, on microbial metabolic activity and biofilm formation [19]. The RMF-induced alterations in metabolic processes directly influence mechanisms that contribute to antibiotic resistance, presenting potential therapeutic advantages in treating infections caused by multidrug-resistant strains. Woroszyło et al. highlighted that bacterial strain diversity leads to variable responses to RMF regarding antibiotic resistance, even within a single species, underscoring the need for studies encompassing a broad spectrum of strain variants [18].

Our findings support this perspective, based on experiments involving 40 phylogenetically diverse colistin-resistant *K. pneumoniae* strains. We observed that the frequency of the RMF (5 Hz vs. 50 Hz) had a differential impact on the MICs of colistin. Specifically, exposure to RMF at 5 Hz resulted in more pronounced MIC reductions across the majority of isolates. In contrast, strains exposed to 50 Hz RMF showed a markedly lower tendency to exhibit changes in colistin MIC values. The mechanisms responsible for the observed reduction in MIC values following RMF exposure are likely complex and multifactorial, involving subtle but biologically meaningful changes in bacterial physiology. Our previous studies have shown that low-frequency RMF (5 and 50 Hz) can induce structural modifications in bacterial cell walls that, although non-lethal, appear to disrupt the integrity of these structures in a way that increases bacterial susceptibility to certain antibiotics [17,19,37,40]. Importantly, RMF exposure alone did not affect the viability of *K. pneumoniae* under our experimental conditions, as also found in the current study. This was confirmed by consistent bacterial growth in control cultures incubated without colistin, both under standard conditions and in the presence of RMF. Specifically, cultures recovered on agar plates exhibited comparable colony morphology and density, while parallel control wells in the broth microdilution assay (MIC COL kit, Diagnostics, Galanta, Slovakia) showed no differences in visible growth between RMF-exposed and non-exposed groups. These observations are consistent with our previous findings on *S. aureus* and *P. aeruginosa*, where RMF alone did not reduce bacterial viability, further supporting the interpretation that the observed MIC reductions were not attributable to nonspecific suppression of bacterial growth [37]. Cell wall perturbations observed in our previous studies may nonetheless render bacteria more susceptible to antimicrobials, particularly those acting on the cell wall, such as β-lactams or colistin [17,19,37,40]. Although the present study focused on planktonic *K. pneumoniae*, additional evidence from our previous research suggests that RMF facilitates antibiotic penetration through biofilm matrices by increasing their porosity and altering saccharide composition. While these findings pertain to a different growth mode and species, they remain mechanistically relevant, as they point to RMF-induced disruptions in membrane-bound transport systems involved in matrix secretion. These disruptions are thought to arise from supramolecular electron excitation at the level of bacterial membranes and cell walls [41,42]. Therefore, such alterations, although observed in a biofilm context, indirectly support our current findings by highlighting a broader principle: RMF can affect key membrane-associated processes, potentially influencing drug susceptibility. This interpretation is further reinforced by GC-MS/MS analyses and SEM-based assessments, which suggest that RMF impacts both planktonic and biofilm-associated cells through related structural and physiological mechanisms [19]. Taken together, these findings point to a synergistic mechanism in which RMF sensitizes multidrug-resistant *K. pneumoniae* strains to colistin by modulating bacterial cell wall structure and enhancing drug–bacteria interactions, without exerting a direct bactericidal effect. The reduction in MIC values observed following 5 Hz RMF exposure appears to stem from non-lethal physiological alterations that compromise bacterial defense mechanisms (particularly those associated with membrane function), thereby facilitating increased antimicrobial efficacy.

Although the present study was performed under strictly controlled in vitro conditions using a compact laboratory RMF generator, the observed reductions in colistin MIC values suggest that RMF may have translational potential as a supportive method to improve antimicrobial efficacy against MDR pathogens. The concept of clinical application of RMF has previously been proposed by Junka et al., who demonstrated that RMF significantly enhanced the in vitro activity of antibiotics and antiseptics against *S. aureus* and *P. aeruginosa* biofilms [43]. Based on their findings, the authors suggested the potential development of RMF-based systems for localized treatment of infected wounds. Complementing these results, Ciecholewska-Juśko et al. showed that RMF enhanced the penetration and antimicrobial efficacy of octenidine against mature biofilms of the same pathogens [19]. Their study further reinforces the idea that RMF could support local antimicrobial strategies, particularly in infections involving biofilm formation. Similarly, our current study provides proof-of-concept evidence that RMF exposure can enhance antimicrobial efficacy against MDR pathogens under controlled in vitro conditions. Despite the strength of our molecular and functional data, however, some limitations of this study must be acknowledged. Translating these effects into clinical practice would require substantial further development. Any future RMF-based intervention would need to involve localized exposure, limited to the site of infection, to minimize systemic effects and ensure safety. To achieve this, dedicated RMF devices would have to be engineered to fit anatomical and pathological contexts, particularly for surface-accessible infections such as chronic wounds. Most importantly, rigorous preclinical evaluation would be necessary, including in vivo safety testing, assessment of local tissue responses, thermal effects, interactions with host cells and microbiota, and overall tolerability. Therefore, while the preliminary in vitro results are promising, we are fully aware that clinical implementation must be preceded by careful validation at every stage of translational development.

## 4. Materials and Methods

### 4.1. K. pneumoniae Strains

Twenty (ESBL)-positive *K. pneumoniae* and twenty (KPC)-positive *K. pneumoniae* strains were used in this study. The isolates were obtained from the Department of Microbiology, Immunology, and Laboratory Medicine at the Pomeranian Medical University in Szczecin collection. The strains were stored at freezer temperature on a tryptone-soy medium (Pol-Aura, Zawroty, Poland). The designed studies did not require the approval of the bioethics committee and were carried out in the department from which the strains were obtained.

The *K. pneumoniae* strains were identified through the MALDI-TOF MS method (Bruker, Berlin, Germany). Additionally, molecular typing was performed using pulsed-field gel electrophoresis (PFGE) to assess their clonal diversity, employing CHEF Bacterial Genomic DNA Plug Kits (Bio-Rad, Marnes-la-Coquette, France). The classification of individual restriction patterns for particular genetic profiles was carried out using the Unweighted Pair Group Method with Arithmetic Mean (UPGMA) method (SAB value = 82.5%) and the Dice coefficient (2.0%). Antimicrobial susceptibility testing (AST) for amoxicillin with clavulanic acid, piperacillin, piperacillin–tazobactam, cefotaxime, ceftazidime, gentamicin, amikacin, meropenem, imipenem, ertapenem, ciprofloxacin, and trimethoprim–sulfamethoxazole was performed using the Kirby–Bauer agar disk diffusion method according to the guidelines European Committee on Antimicrobial Susceptibility Testing (EUCAST) [44]. The presence of ESBLs was confirmed with the double disk diffusion method (DDST). The presence of the *bla*_KPC_ gene was confirmed by Real-Time PCR XpertCarbaR (Cepheid, Solna, Sweden).

### 4.2. Rotating Magnetic Field Generator and Magnetic Exposure

**Figure 3:**
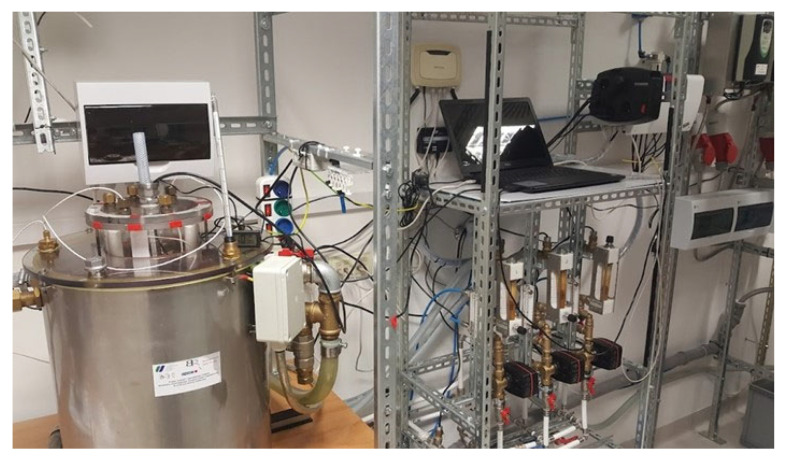
Rotating magnetic field generator.

The exposure to the RMF was carried out using self-designed RMF bioreactors, described in our previous works and adopted for purposes of this research [15,18]. One of the bioreactors was operated with the active RMF generator, while the second served as the control setup (without RMF). Each RMF bioreactor used a 3-phase, 4-pole RMF generator with an inner diameter of 16 cm and a height of 20 cm (Figure 3; see also video demonstration: https://www.youtube.com/watch?v=S1I6ZAZymLA, accessed on 12 May 2025).

The generator comprised 12 subunits, each containing three sets of coils. A Unidrive M200 inverter (Control Techniques, Nidec Industrial Automation, Poznan, Poland) was utilized to vary the frequency of the alternating current supplied to the RMF generator, ranging from 5 to 50 Hz. Temperature stability within the RMF reactor chamber was maintained by a powered heating and cooling system equipped with probes that accurately measured and reported temperature variations within ±1.0 °C. During RMF exposure, airflow was regulated to ensure optimal temperature distribution within the bioreactor, operating at 2 L/min, 35.0 °C, and 60% relative humidity.

The duration of exposure to RMF in all experimental variants was 12 h, consistent with the protocol applied in previous studies by Woroszyło et al., where this exposure time was standardized to ensure reproducibility and comparability of RMF-induced effects on microbial susceptibility [37,40]. The incubation temperature of 35 °C was selected to support bacterial viability during prolonged exposure and is consistent with the guidelines established by EUCAST for antimicrobial susceptibility testing.

### 4.3. Evaluation of the Effect of RMF on Susceptibility to Colistin

In the first stage of the study, in accordance with EUCAST guidelines, colistin susceptibility was determined using the microdilution method in broth using the MIC COL kit (Diagnostics, Galanta, Slovakia). The determination was performed on a titration plate with varying colistin concentrations, covering eight wells with microorganisms, of which 7 were test samples and 1 was a control sample. The colistin concentrations increased exponentially, from a minimum of 0.25 mg/L to a maximum of 16 mg/L.

During the second stage of the study, experimentally, only for comparison with the dilution method, E-test strips with a gradient of colistin concentrations ranging from 0.016 to 256 mg/L were used (BioMerieux, Marcy-l’Étoile, France). Antibiotic susceptibility was evaluated in accordance with EUCAST guidelines (from 2025 and 2017, respectively). A minimum inhibitory concentration (MIC) of ≤2 mg/L indicated that the strains were susceptible to colistin, whereas a MIC of >2 mg/L was classified as resistant. The colistin-susceptible *K. pneumoniae* strain BAA 1705 served as a control in this study.

## 5. Conclusions

The *K. pneumoniae* strains tested exhibited the production of ESBLs or KPC and demonstrated resistance to the majority of antibiotics. All strains were resistant to colistin. The application of 5 Hz RMF proved to be more effective than 50 Hz, as a greater number of strains showed a reduction in MIC values at this frequency, although these values remained within the resistant category. The results suggest the potential of RMF as a method supporting the antibacterial action of antibiotics, in this case, colistin. However, further preclinical studies are necessary to assess the safety, tolerance, and impact of RMF on tissues and microflora before clinical implementation.

## Figures and Tables

**Table 1:** Drug susceptibility of (ESBL)-positive *K. pneumoniae* strains.

Antibiotic	Antibiotic Concentration (µg)	Resistant *n* (%)	Susceptibility *n* (%)
Piperacillin	30	20 (100.0)	0 (0.0)
Piperacillin/tazobactam	30–6		
Amoxicillin with clavulanic acid	20–10		
Ceftazidime	10		
Cefotaxime	30		
Amikacin	30	8 (40.0)	12 (60.0)
Gentamicin	10	9 (45.0)	11 (55.0)
Ciprofloxacin	5	6 (30.0)	14 (70.0)
Trimethoprim/sulfamethoxazole	1.25–23.75	7 (35.0)	13 (65.0)
Ertapenem	10	0 (0.0)	20 (100.0)
Meropenem	10		
Imipenem	10		

**Table 2:** Drug susceptibility of (KPC)-positive *K. pneumoniae* strains.

Antibiotic	Antibiotic Concentration (µg)	Resistant *n* (%)	Susceptibility *n* (%)
Piperacillin	30	20 (100.0)	0 (0.0)
Piperacillin/tazobactam	30–6		
Amoxicillin with clavulanic acid	20–10		
Ceftazidime	10		
Cefotaxime	30		
Meropenem	10		
Imipenem	10		
Ertapenem	10		
Amikacin	30	15 (75.0)	5 (25.0)
Gentamicin	10	16 (80.0)	4 (20.0)
Ciprofloxacin	5	12 (60.0)	8 (40.0)
Trimethoprim/sulfamethoxazole	1.25–23.75	17 (85.0)	3 (15.0)

**Table 3:** MIC Colistin for (ESBL)-positive and (KPC)-positive *K. pneumoniae* strains after 5 Hz RMF exposure.

Mechanism	MIC COL Kit Control (mg/L)	MIC COL Kit + 5 Hz (mg/L)	Number of Strains *n* (%)	Effect After RMF Exposure	E-Test Control (mg/L)	E-Test + 5 Hz (mg/L)	Number of Strains *n* (%)	Effect After RMF Exposure
ESBLs	4	4	11 (55.0)	↔	4	4	11 (55.0)	↔
	8	8			8	8		
	16	16			16	16		
	8	4	9 (45.0)	↓	6	4	9 (45.0)	↓
	8	4			8	4		
	16	8			16	8		
	16	4			16	4		
KPC	4	4	10 (50.0)	↔	4	4	10 (50.0)	↔
	8	8			8	8		
	16	16			16	16		
	8	4	10 (50.0)	↓	8	4	10 (50.0)	↓
	16	8			16	8		
	16	4			16	4		
	4	4			4	4		

Legend: ↔—no difference; ↓—lower MIC value.

**Table 4:** MIC Colistin for (ESBL)-positive and (KPC)-positive *K. pneumoniae* strains after 50 Hz RMF exposure.

Mechanism	MIC COL Kit Control (mg/L)	MIC COL Kit + 5 Hz (mg/L)	Number of Strains *n* (%)	Effect After RMF Exposure	E-Test Control (mg/L)	E-Test + 5 Hz (mg/L)	Number of Strains *n* (%)	Effect After RMF Exposure
ESBLs	4	4	12 (60.0)	↔	4	4	10 (50.0)	↔
	8	8			8	8		
	16	16			16	16		
	8	4	7 (35.0)	↓	6	4	5 (25.0)	↓
	16	8			16	8		
	8	16	1 (5.0)	↑	6	8	5 (25.0)	↑
					8	16		
KPC	4	4	11 (55.0)	↔	4	4	11 (55.0)	↔
	8	8			8	8		
	16	16			16	16		
	8	4	8 (40.0)	↓	8	4	8 (40.0)	↓
	16	8			16	8		
	4	8	1 (5.0)	↑	4	8	1 (5.0)	↑

Legend: ↔—no difference; ↓—lower MIC value; ↑—higher MIC value.

## Data Availability

The original contributions presented in this study are included in the article and Supplementary Materials. Further inquiries can be directed to the corresponding authors.

## Supplementary Materials

The following supporting information can be downloaded at: https://www.mdpi.com/article/10.3390/ijms26178281/s1.

## Abbreviations

The following abbreviations are used in this manuscript:

ESBLs	Extended-spectrum beta-lactamases
KPC	*Klebsiella pneumoniae* carbapenemase
MDR	Multidrug-resistant
ECDC	European Centre for Disease Control
XDR	Extended multidrug-resistant
PDR	Pandrug-resistant
NDM	New Delhi Metallo-beta-lactamases
CHDL	Carbapenem-hydrolyzing class D beta-lactamases
MRSA	Methicillin-resistant *Staphylococcus aureus*
PFGE	Pulsed-field gel electrophoresis
AST	Antimicrobial susceptibility testing
EUCAST	European Committee on Antimicrobial Susceptibility Testing
DDST	Double disk diffusion method
