# Supplementary material for: Evaluation of Colistin Susceptibility of Klebsiella pneumoniae Strains Exposed to Rotating Magnetic Field
Source: Int J Mol Sci. 2025 Aug 26;26(17):8281. doi: 10.3390/ijms26178281 (PMC12428290; doi:10.3390/ijms26178281)
Supplement: Supplementary file 1 [file ijms-26-08281-s001.zip › ijms-3807488-supplementary.pdf]

Tab.S1 Summary table with drug susceptibility data and effects of RMF on *K. pneumoniae* strains.

Strainnumber	Mechanism of resistance	Antibiotics (Kirby-Bauer method)												MIC colistin					
		AMC	PIP	TZP	CAZ	CTX	AN	GE	CIP	SXT	MEM	IMP	ERT	MIC COL kit (control) (mg/L)	MIC COL kit + 5 Hz (mg/L)	MIC COL kit + 50 Hz (mg/L)	E- test (control) (mg/L)	E-test + 5 Hz (mg/L)	E-test + 50 Hz (mg/L)
1	ESBL													16.0 ± 0.0	16.0 ± 0.0	8.0 ± 0.0	16.0 ± 0.0	16.0 ± 0.0	16.0 ± 0.0
2														4.0 ± 0.0	4.0 ± 0.0	4.0 ± 0.0	4.0 ± 0.0	4.0 ± 0.0	4.0 ± 0.0
3														8.0 ± 0.0	4.0 ± 0.0	4.0 ± 0.0	6.0± 0.0	8.0 ± 0.0	4.0 ± 0.0
4														4.0 ± 0.0	4.0 ± 0.0	4.0 ± 0.0	4.0 ± 0.0	4.0 ± 0.0	4.0 ± 0.0
5														8.0 ± 0.0	4.0 ± 0.0	8.0 ± 0.0	8.0 ± 0.0	8.0 ± 0.0	4.0 ± 0.0
6														16.0 ± 0.0	8.0 ± 0.0	8.0 ± 0.0	16.0 ± 0.0	16.0 ± 0.0	8.0 ± 0.0
7														8.0 ± 0.0	4.0 ± 0.0	8.0 ± 0.0	6.0 ± 0.0	8.0 ± 0.0	4.0 ± 0.0
8														16.0 ± 0.0	16.0 ± 0.0	16.0 ± 0.0	16.0 ± 0.0	16.0 ± 0.0	16.0 ± 0.0
9														8.0 ± 0.0	4.0 ± 0.0	8.0 ± 0.0	6.0 ± 0.0	8.0 ± 0.0	4.0 ± 0.0
10														8.0 ± 0.0	8.0 ± 0.0	8.0 ± 0.0	8.0 ± 0.0	8.0 ± 0.0	8.0 ± 0.0
11														16.0 ± 0.0	8.0 ± 0.0	8.0 ± 0.0	16.0 ± 0.0	16.0 ± 0.0	8.0 ± 0.0
12														8.0 ± 0.0	8.0 ± 0.0	8.0 ± 0.0	8.0 ± 0.0	8.0 ± 0.0	8.0 ± 0.0
13														16.0 ± 0.0	16.0 ± 0.0	16.0 ± 0.0	16.0 ± 0.0	16.0 ± 0.0	16.0 ± 0.0
14														16.0 ± 0.0	8.0 ± 0.0	8.0 ± 0.0	16.0 ± 0.0	16.0 ± 0.0	8.0 ± 0.0
15														8.0 ± 0.0	8.0 ± 0.0	8.0 ± 0.0	8.0 ± 0.0	8.0 ± 0.0	8.0 ± 0.0
16														8.0 ± 0.0	4.0 ± 0.0	8.0 ± 0.0	6.0 ± 0.0	8.0 ± 0.0	4.0 ± 0.0
17														8.0 ± 0.0	8.0 ± 0.0	8.0 ± 0.0	8.0 ± 0.0	8.0 ± 0.0	8.0 ± 0.0
18														4.0 ± 0.0	4.0 ± 0.0	4.0 ± 0.0	4.0 ± 0.0	4.0 ± 0.0	4.0 ± 0.0
19														16.0 ± 0.0	4.0 ± 0.0	8.0 ± 0.0	16.0 ± 0.0	16.0 ± 0.0	4.0 ± 0.0
20														8.0 ± 0.0	8.0 ± 0.0	16.0 ± 0.0	8.0 ± 0.0	8.0 ± 0.0	8.0 ± 0.0
21	KPC													4.0 ± 0.0	4.0 ± 0.0	4.0 ± 0.0	4.0 ± 0.0	4.0 ± 0.0	4.0 ± 0.0
22														16.0 ± 0.0	8.0 ± 0.0	8.0 ± 0.0	16.0 ± 0.0	8.0 ± 0.0	8.0 ± 0.0
23														4.0 ± 0.0	4.0 ± 0.0	8.0 ± 0.0	4.0 ± 0.0	4.0 ± 0.0	8.0 ± 0.0
24														16.0 ± 0.0	8.0 ± 0.0	16.0 ± 0.0	16.0 ± 0.0	8.0 ± 0.0	16.0 ± 0.0
25														16.0 ± 0.0	8.0 ± 0.0	8.0 ± 0.0	16.0 ± 0.0	8.0 ± 0.0	8.0 ± 0.0
26														4.0 ± 0.0	4.0 ± 0.0	4.0 ± 0.0	4.0 ± 0.0	4.0 ± 0.0	4.0 ± 0.0
27														8.0 ± 0.0	4.0 ± 0.0	4.0 ± 0.0	8.0 ± 0.0	4.0 ± 0.0	4.0 ± 0.0
28														8.0 ± 0.0	8.0 ± 0.0	4.0 ± 0.0	8.0 ± 0.0	8.0 ± 0.0	4.0 ± 0.0
29														4.0 ± 0.0	4.0 ± 0.0	4.0 ± 0.0	4.0 ± 0.0	4.0 ± 0.0	4.0 ± 0.0
30														8.0 ± 0.0	8.0 ± 0.0	8.0 ± 0.0	8.0 ± 0.0	8.0 ± 0.0	8.0 ± 0.0
31														8.0 ± 0.0	4.0 ± 0.0	8.0 ± 0.0	8.0 ± 0.0	4.0 ± 0.0	8.0 ± 0.0
32														16.0 ± 0.0	4.0 ± 0.0	8.0 ± 0.0	16.0 ± 0.0	4.0 ± 0.0	8.0 ± 0.0
33														8.0 ± 0.0	8.0 ± 0.0	8.0 ± 0.0	8.0 ± 0.0	8.0 ± 0.0	8.0 ± 0.0
34														8.0 ± 0.0	4.0 ± 0.0	4.0 ± 0.0	8.0 ± 0.0	4.0 ± 0.0	4.0 ± 0.0
35														16.0 ± 0.0	8.0 ± 0.0	16.0 ± 0.0	16.0 ± 0.0	8.0 ± 0.0	16.0 ± 0.0

36													16.0 ± 0.0	4.0 ± 0.0	8.0 ± 0.0	16.0 ± 0.0	4.0 ± 0.0	8.0 ± 0.0
37													8.0 ± 0.0	8.0 ± 0.0	8.0 ± 0.0	8.0 ± 0.0	8.0 ± 0.0	8.0 ± 0.0
38													8.0 ± 0.0	4.0 ± 0.0	4.0 ± 0.0	8.0 ± 0.0	4.0 ± 0.0	4.0 ± 0.0
39													4.0 ± 0.0	4.0 ± 0.0	4.0 ± 0.0	4.0 ± 0.0	4.0 ± 0.0	4.0 ± 0.0
40													16.0 ± 0.0	16.0 ± 0.0	16.0 ± 0.0	16.0 ± 0.0	16.0 ± 0.0	16.0 ± 0.0
